# Promoter hypermethylation of tumor suppressor genes correlates with tumor grade and invasiveness in patients with urothelial bladder cancer

**DOI:** 10.1186/2193-1801-3-178

**Published:** 2014-04-05

**Authors:** Shumaila M Bilgrami, Sohail A Qureshi, Shahid Pervez, Farhat Abbas

**Affiliations:** Office of Research and Graduate Studies, Aga Khan University, Stadium Road, Karachi, 74800 Pakistan; Department of Biology, Syed Babar Ali School of Science and Engineering, Lahore University of Management Sciences, Sector-U, D.H.A., Lahore, 54792 Pakistan; Department of Microbiology and Pathology, Aga Khan University, Stadium Road, Karachi, 74800 Pakistan; Department of Surgery, Aga Khan University, Stadium Road, Karachi, 74800 Pakistan

**Keywords:** Promoter methylation, Tumor suppressor genes, Paraffin embedded tissue, Plasma, Urothelial cell carcinoma of bladder

## Abstract

**Purpose:**

To investigate the promoter methylation status at selected loci which encode for key proteins involved in apoptosis, DNA repair, cell cycle control and progression in urothelial cell carcinoma of bladder and compare the findings from tissue samples with that of plasma.

**Methods:**

Total genomic DNA was isolated from 43 non-muscle invasive (low grade) and 33 muscle invasive (high grade) urothelial bladder cancer samples along with 10 control cases of normal bladder mucosa. Promoter methylation status was investigated for *RASSF1A*, *APC*, *MGMT*, *CDKN2A* and *CDKN2B* genes using real-time methylation-specific PCR with SYBR® green. Plasma samples from 16 patients with muscle invasive high grade bladder cancer were also subjected to similar analyses.

**Results:**

Promoter hypermethylation was frequently observed in *RASSF1A*, *APC* and *MGMT* gene promoters (*p-value < 0.001*). The methylation was more prominent in the muscle invasive high grade bladder cancer when compared to non-muscle invasive low grade group (*p-value < 0.001*) and normal bladder mucosa (*p-value < 0.05*). The RNA expression of *RASSF1A*, *APC* and *MGMT* was also found to be decreased in the muscle-invasive high grade bladder cancer when compared to the non muscle invasive low grade group (*p-value < 0.05*)*. RASSF1A*, *MGMT* and *CDKN2A* showed comparable results when data from 16 plasma samples was compared to the corresponding tissue samples.

**Conclusion:**

Our results suggest that epigenetic silencing of *RASSF1A*, *APC* and *MGMT* genes is strongly associated with invasive high grade urothelial bladder cancer. Thus, status of promoter methylation has the potential to serve as valuable tool for assessing aggressiveness of urothelial cell carcinoma of bladder.

**Electronic supplementary material:**

The online version of this article (doi:10.1186/2193-1801-3-178) contains supplementary material, which is available to authorized users.

## Background

Urinary bladder cancer represents the second most frequent urologic cancer worldwide which accounts for 3.3% of newly diagnosed cancer cases and 2.1% of cancer deaths. Globally, the incidence of bladder cancer is highest among North American and Western European populations (<16.2 per 100,000); in comparison Eastern Europe & Asian countries have relatively lower rates (<1.7 per 100,000). Alarmingly, Pakistan carries a heavy burden of disease among all South Central Asian countries with mortality rate of 3.8 and 1.1 per 100,000 in males and females, respectively (IARC 2013).

At present, cystoscopy followed by histological examination serve as the gold standard for making the initial diagnosis and monitoring progression of bladder tumors. Although cystoscopy provides valuable information regarding tumor status, it involves an invasive procedure which is not very cost effective. In view of this, there is a dire need to identify robust genetic and/or epigenetic markers that can serve as reliable indicators of disease. Over the past two decades a number of biomarkers such as nuclear matrix protein 22 (NMP22), fibrin degradation product (Fibrin/FDP), bladder tumor antigen (BTA), high molecular weight carcinoembryonic antigen and mucin have been identified and approved by FDA for monitoring and screening of bladder cancers but initial enthusiasm for their clinical utility waned quickly because each of them lacked specificity, reproducibility as well as sensitivity (Ludwig and Weinstein 2005).

DNA methylation negatively influences transcription and plays a pivotal role in shaping the epigenome to ascertain accurate temporal and spatial expression of genes during development. It occurs in distinct regions of promoters where the density of the dinucleotide CpG is higher than would be expected in random sequence. These so called “CpG islands” are found in over 60% of the human genes. The process of converting an unmodified CpG into one which is methylated at the C5 position of cytosine is catalyzed by DNA methyltransferases (DNMT). DNMT1 uses hemi-methylated CpG resides as substrate and fully methylates them. In contrast, DNMT3 is capable of methylating unmodified CpG dinucleotides *de novo*. Since DNA hypermethylation serves as a powerful mechanism that turns off gene transcription any unprogrammed changes in the epigenome – be it hypermethylation of a tumor suppressor genes (TSGs) or demethylation of oncogenes – are likely to culminate in a disease state. Indeed, silencing of tumor suppressor genes due to CpG hypermethylation has been well documented in different types of cancers. In bladder cancer, expression and function of a number of TSGs including *PTCH*, *TSC1*, *RB1*, *PTEN*, *p53*, *DAPK*, *FHIT*, *CDH1*, *CDKN2B*, *CDKN2A*, *APC*, *RASSF1A* and *MGMT* are known to be impacted either by physical changes in the sequence of DNA or by un-programmed DNA methylation (Cairns 2007; Knowles 2007).

In this study we have investigated the DNA methylation status of a panel of tumor suppressor genes that include *RASSF1A*, *APC*, *MGMT*, *CDKN2A* and *CDKN2B* using formalin fixed paraffin embedded (FFPE) biopsies obtained from individuals suffering from non- muscle invasive and muscle invasive urothelial cell carcinoma of bladder from Pakistan. Normal bladder mucosa/benign urologic disease samples were used as controls. These genes were selected because their respective products influence cell cycle control, apoptosis and DNA repair, and because they have been found to be epigenetically silenced in many human neoplasms.

## Results

Our study included 76 patients with transitional cell carcinoma of bladder from which 43 were non-muscle invasive tumors (pTa-T1) and 33 muscle invasive tumors (≥pT2). Transitional cell carcinoma was low grade (including papillary urothelial neoplasm of low malignant potential) in 43 patients and high grade in 33 patients. The median age of patients was 64 years in non-invasive low grade cancer group and 61 years in invasive high grade bladder cancer. Methylation analyses were also carried out in 10 patients with benign urologic disease as control group with a median age of 49 years. Details of the individuals whose tissues were used in this study are listed in Table 1.

**Table 1:** **Clinicopathological parameters of normal and bladder Cancer FFPE samples**

Parameter	Low grade urothelial cancer	High grade urothelial cancer	Normal bladder mucosa
**Number of patients**	43	33	10
**Males**	37(86%)	26(78%)	05
**Females**	06(14%)	07(21%)	05
**Age**			
Median (Inter-quartile range)	64(50-73)	61(53-68)	49(40-59)
**Pathologic stage**			
pTa-pT1	43		
≥pT2		33	

(pTa: Non-invasive papillary carcinoma, pT1: Tumor invades sub epithelial connective tissue, pT2: Tumor invades muscle, pT3: Tumor invades perivesical tissue, pT4: Tumor invades any of the following: prostate, uterus, vagina, pelvic wall, abdominal wall).

Genomic DNA from the 76 FFPE diseased as well as 10 control tissues was isolated, modified by bisulfite reagent and subsequently used as template for carrying out methylation-specific real-time PCR analysis using pairs of gene-specific primer sets. This revealed that the prevalence of promoter hypermethylation in all five tumor suppressor genes was higher in the muscle invasive high grade urothelial bladder cancer as compared to the non-muscle invasive low grade group (Table 2).

**Table 2:** **Frequency of hypermethylation of tumor suppressor gene loci in urothelial bladder cancer samples**

Tumor suppressor gene	Low grade urothelial carcinoma of urinary bladder (TCC) n = 43	High grade urothelial carcinoma of urinary bladder (TCC) n = 33
***RASSF1A***	49% (21/43)	82% (27/33)
***APC***	51% (22/43)	97% (32/33)
***MGMT***	93% (41/43)	94% (31/33)
***CDKN2A***	19% (8/43)	24% (8/33)
***CDKN2B***	37% (16/43)	57% (19/33)

Out of the five tumor suppressor genes studied in the normal bladder mucosa, muscle invasive and non-muscle invasive urothelial bladder cancer, the promoter hypermethylation at *RASSF1A*, *APC* and *MGMT* was statistically significant (p < 0.001; Table 3). Unexpectedly, *RASSF1A* promoter was also found to be hypermethylated in 5 (2 females and 3 males) out of 10 normal bladder mucosa samples.

**Table 3:** **Normalized index of methylation (NIM)% and corresponding p-values**

	***RASSF1A***	***APC***	***MGMT***	***CDKN2A***	***CDKN2B***
**Invasive high grade urothelial bladder cancer (n = 33)**					
Mean	40	6.1	9	0.09	0.58
Range	0-270	0-50	0-96	0-2	0-6
**Non-invasive low grade urothelial bladder cancer (n = 43)**					
Mean	2	2	2.3	0.4	0.1
Range	0-49	0-43	0-41	0-8	0-2
**Normal bladder (n = 10)**					
Mean	2	0	0	1	2
Range	0-12	0	0-4	0-16	0-1
*p-value	**<0.001**	**<0.001**	**<0.001**	**0.748**	**0.114**
**Plasma samples (n = 16)**					
Mean	24.9	0.035	16.2	1.5	5.9
Range	3-125	0-4.4	0.1-111.7	0-16.3	0.1-18.9
**p-value	**0.539**	<0.001	**0.524**	**0.215**	<0.01

*Kruskal Wallis Test **Paired *T*-Test (p-value<0.01).

### Comparison of normalized index of methylation

Normalized index of methylation (NIM) for all the tumor suppressor genes in the cancer and control group was calculated. The NIM serves as an index of the percentage of bisulfite converted input copies of DNA that are fully methylated at the primer binding sites. However, it is important to note that the NIM maybe >1 if the copies of *Actin* are deleted relative to the gene of interest, or copies of the gene of interest are gained relative to *Actin* in any given sample.

**Figure 1:**
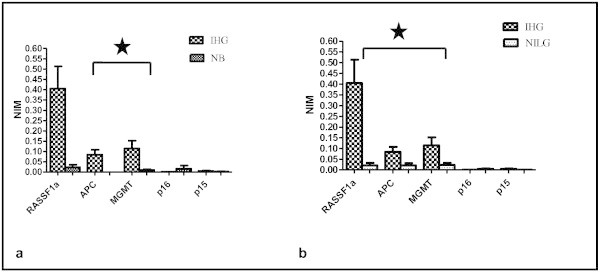
**Comparison of Normalized index of methylation (NIM) for the tumor suppressor genes. a** Between normal bladder mucosa (NB) and muscle invasive high grade (IHG) urothelial bladder cancer (*p-value for *APC* and *MGMT* was **<**0.001) **b** Between muscle invasive high grade (IHG) and non-muscle invasive low grade (NILG) urothelial bladder cancer (*p-value for *RASSF1A*, *APC* and *MGMT* was <0.001 each-*Mann–Whitney-*U* Test).

When the mean normalized index of methylation of the genes was compared between non- muscle invasive low grade and muscle invasive high grade urothelial bladder cancer, the degree of hypermethylation was more prominent in the muscle invasive high grade group for *RASSF1A*, *APC* and *MGMT* (*p < 0.001*). However, only *APC* and *MGMT* showed significance (*p < 0.001*) in the muscle invasive high grade bladder cancer when it was compared with the normal bladder mucosa (Figure 1). Interestingly, none of the tumor suppressor genes reached statistical significance when NIM was compared between normal bladder mucosa and non-muscle invasive low grade bladder cancer (Table 4).

**Table 4:** **Comparison of NIM and corresponding p-values**

	***RASSF1A***	***APC***	***MGMT***	***CDKN2A***	***CDKN2B***
**NB vs. NILG	0.03	0.04	1.00	0.61	0.17
**NB vs. IHG**	0.04	<0.001*	<0.01*	0.58	1.00
**NILG vs. IHG**	<0.001*	<0.001*	<0.001*	0.72	0.05

*Mann–Whitney-*U* Test (p-value < 0.01, after correction for multiple testing); **NB = Normal bladder mucosa, sNILG = Non-muscle invasive low grade urothelial bladder cancer; IHG = Muscle invasive high grade urothelial bladder cancer).

### Change in mRNA expression

**Figure 2:**
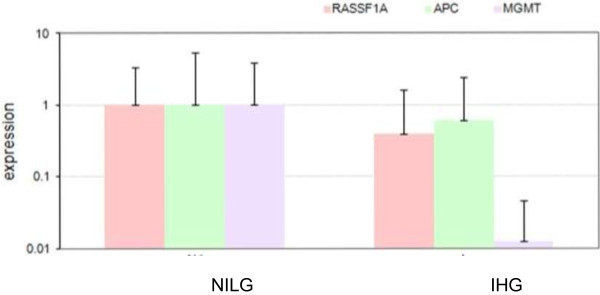
**RNA Expression of tumor suppressor genes** ***RASSF1A*** **,** ***APC*** **and** ***MGMT*** **in non muscle invasive low grade (NILG) and muscle invasive high grade (IHG) urothelial cell carcinoma of bladder.** Total RNA was extracted from formalin fixed and paraffin embedded samples, was reverse transcribed and amplified using primers against *RASSF1a*, *APC* and *MGMT* to determine any difference in the expression of RNA. (p-value for *RASSF1A*, *APC* and *MGMT* was <0.05 each-when Wilcoxon signed rank test was applied).

NIM was found to be higher for *RASSF1A*, *APC* and *MGMT* in muscle invasive high grade urothelial bladder cancer as compared to the non muscle invasive low grade cancer. In order to determine any difference in the gene expression between the two groups, total RNA was extracted from formalin fixed and paraffin embedded samples, was reverse transcribed and amplified using specific primers. The mRNA expression of *RASSF1A*, *APC* and *MGMT* was found to be decreased in the muscle invasive high grade urothelial cell carcinoma of bladder as compared to the non- muscle invasive low grade group (Figure 2), and this finding was statistically significant (p-value < 0.05) when Wilcoxon signed rank test was applied.

### Association between biopsy and plasma samples

In the next step, to assess whether there is an association between the promoter methylation patterns detected in tissue samples with those in blood samples, promoter hypermethylation at the tumor suppressor genes was also determined in sixteen plasma samples from patients with invasive high grade urothelial bladder cancer. When the results were statistically analyzed using paired-*T* test, *RASSF1A*, *MGMT* and *CDKN2A* showed comparable results in the cancer tissue and plasma samples. Whereas, *APC* (*p < 0.01*) and *CDKN2B* (*p < 0.05*) showed significant difference between the two.

### Survival analysis

**Figure 3:**
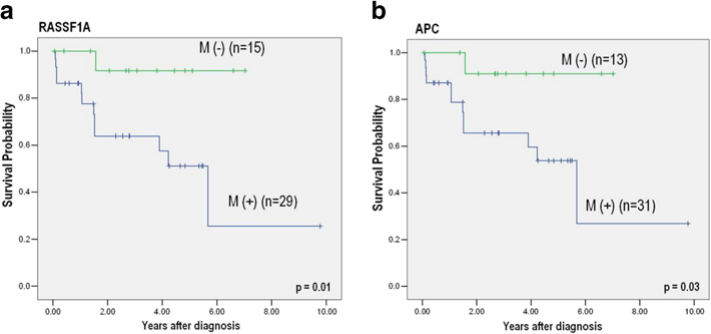
**Association of patients’ survival and promoter methylation status by Kaplan-Meier method. a** survival curves by methylation status of *RASSF1A* (Number of censored cases with and without methylation were 17 and 14, respectively) **b** survival curves by methylation status of *APC* (Number of censored cases with and without methylation were 19 and 12, respectively).

By Kaplan-Meier analysis, the methylation status of genes did not show any significance in correlation with the recurrence-free interval. However, a pattern was observed with worse recurrence free survival in methylation positive urothelial cell carcinoma of bladder. When the overall survival was correlated with the methylation status of candidate tumor suppressor genes, it was found to be significantly shorter in patients with *RASSF1A* and *APC* methylation positive tumor (Figure 3).

## Discussion

In the present study, we have investigated the promoter methylation status of candidate tumor suppressor genes namely *RASSF1A*, *APC*, *MGMT*, *CDKN2A* and *CDKN2B* in normal bladder mucosa, invasive high grade and non-invasive low grade urothelial bladder cancer using formalin fixed paraffin embedded tissue and plasma samples.

Our results demonstrate that the prevalence of promoter methylation was higher in invasive high grade urothelial bladder cancer as compared to the non-invasive low grade. Out of the five genes studied the prevalence of hypermethylation was 82%, 97% and 94% for *RASSF1A*, *APC* and *MGMT*, respectively. Interestingly, the findings from formalin fixed paraffin embedded samples were comparable to the plasma samples of invasive high grade bladder cancer patients for *CDKN2A*, *MGMT* and *RASSF1A*.

The *RASSF1* (Ras association domain family 1) family represents a class of Ras effector proteins that have tumor suppressor properties. *RASSF1A* acts as a putative tumor suppressor gene located on 3p21 and may serve as an effector that mediates the apoptotic effects by binding Ras in a guanosine triphosphate-dependent manner (Hesson et al. 2007). Studies have shown that as compared to the classic mutations, promoter hypermethylation serve as major mechanism of gene silencing in *RASSF1A*.

Promoter methylation induced silencing of *RASSF1A* has been found to be associated with multiple human cancers (Bhagat et al. 2012; Jiang et al. 2012; Koutsimpelas et al. 2012; Munoz et al. 2012; Jeronimo et al. 2004). Previous studies have shown that *RASSF1A* methylation was detectable in body fluids including plasma, nipple aspirate, sputum, urine and bronchoalveolar lavage (Dulaimi et al. 2004; Krassenstein et al. 2004; Topaloglu et al. 2004; Belinsky et al. 2005; Hoque et al. 2006).

In bladder cancer, *RASSF1A* methylation has been linked to high grade bladder tumors and poor prognosis (Gao et al. 2012; Meng et al. 2012; Maruyama et al. 2001). We also found *RASSF1A* methylation in five out of ten normal bladder mucosa samples, which is in agreement with other studies. But this finding would raise questions about its utility as a biomarker due to low specificity; hence, studies on larger sample size are needed to address this issue.

Mutations in *APC* (Adenomatous polyposis coli) gene are known to be associated with Familial adenomatous polyposis coli and colorectal cancer. *APC* protein acts as a negative regulator of WNT/β-catenin signaling pathway as it promotes the proteolytic degradation of β-catenin in Wnt signaling pathway. It also plays a role in cell migration and adhesion, apoptosis and transcriptional activation (Klaus and Birchmeier 2008).

As an interesting finding hypermethylation of *APC* gene promoter was more prominent in invasive high grade urothelial bladder cancer as compared to noninvasive low grade one which is also in line with the previous studies correlating *APC* methylation with tumor grade, stage and muscle invasion (Berrada et al. 2012; Hoque et al. 2006).

*MGMT* gene encodes an enzyme called O-6-Methylguanine DNA methyltransferase which removes a carcinogenic DNA lesion, O (6)-alkyl-guanine. The promoter methylation at *MGMT* has been reported in many human cancers but found to be less frequent in bladder cancer. (Bhagat et al. 2012; Koutsimpelas et al. 2012; Liu et al. 2011; Yates et al. 2007) Our study shows a prominent *MGMT* hypermethylation in the invasive high grade bladder cancer as well as the non-invasive low grade cancer and the findings were correlated in the plasma samples. However, the the methylation index was higher in the invasive high grade urothelial bladder cancer.

Impaired regulation of cell cycle control is an important event in tumorigenesis. The regulation of cell cycle involves multiple check points including cyclins, cyclin dependent kinase enzymes and cyclin dependent kinase inhibitors. Cyclin dependent kinase inhibitor 2A and 2B, *p16* and *p15* respectively, act as negative regulators of cell cycle progression and thus are potential tumor suppressors *p16*/cyclin-dependent kinase inhibitor *2A* (*CDKN2A*) encodes splice variants and is involved in sequestering MDM2 so *p53* gets stabilized. It inhibits enzyme CDK4 kinase, hence inhibits progression of cell cycle from G1 phase.

Studies on *CDKN2A/p16* promoter methylation in bladder cancer have shown varying results reporting the frequency of methylation ranging from 7-60% (Chan, Chan et al. 2002; Dominguez et al. 2002; Chang et al. 2003; Catto et al. 2005). In our study, the overall methylation frequency for *CDKN2A*/*p16* was low in both invasive high grade (24%) and non-invasive low grade (19%) urothelial bladder cancer with a very low normalized index of methylation. However, the results were found to be analogous when the *CDKN2A*/*p16* methylation found in paraffin embedded samples was compared to the plasma samples.

Like *p16*, *p15*/cyclin-dependent kinase inhibitor 2B (*CDKN2B*) also encodes a cyclin dependent kinase inhibitor which inhibits CDK4 or CDK6 kinases and prevents the cell cycle progression from G1 phase. Significant promoter hypermethylation has been reported for both *CDKN2A* and *CDKN2B* in cervical cancer patients of North Indian origin (Jha et al. 2012).

Promoter methylation at *CDKN2B/p15* is not frequent in bladder cancer, as previous studies have reported a methylation frequency ranging from 0-13% (Le Frere-Belda et al. 2001). Our results have shown that *CDKN2B* promoter methylation was slightly higher than *CDKN2A* but the findings of *CDKN2B* methylation from paraffin embedded sample were not comparable to the plasma samples.

## Conclusion

To summarize, our study demonstrates that promoter hypermethylation in tumor suppressor genes plays an important role in bladder cancer development and progression which is evident from the finding that methylation of some tumor suppressor genes associates well with tumor grade, invasiveness and patient’s survival.

Although much has been published in this regard but data from developing world is lacking. This study, though involving some preliminary results on plasma samples, is the first of its kind on a south central Asian population. If the findings are validated using larger number of samples, it could serve as a rapid tool for assessing aggressiveness of bladder cancer in the Pakistani population.

However, to select an ideal gene panel that is specific for bladder cancer still remains a critical issue for designing the most useful panel of epigenetic biomarkers that can come into clinical practice for an early diagnosis or monitoring the progression of disease.

## Methods

### Samples

**Figure 4:**
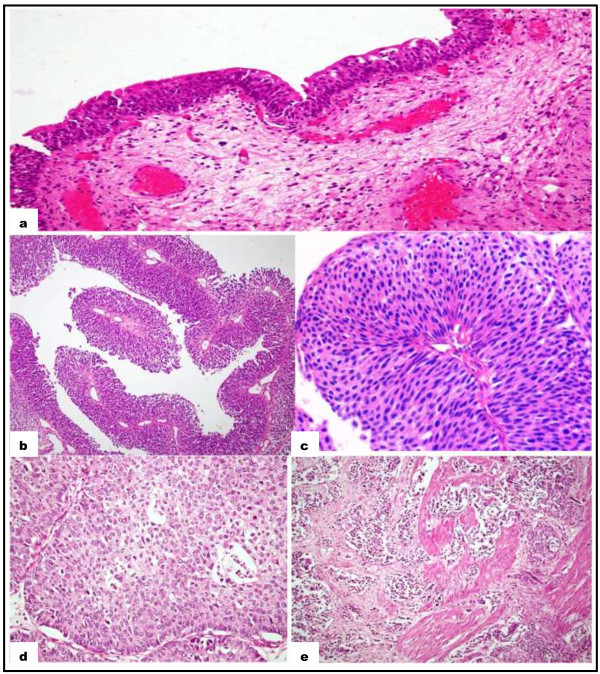
**H & E Staining. a** Normal urothelium, **b** and **c** Non muscle invasive and low grade Urothelial cell carcinoma of bladder 10X and 20X respectively, **d** High grade Urothelial cell carcinoma of bladder, **e** Muscle invasion in high grade Urothelial cell carcinoma of bladder.

This study was approved by the Ethical Review Committee of The Aga Khan University and all samples used or collected followed the approved protocol. Source of bladder cancer tissues were the 76 formalin fixed paraffin embedded blocks prepared from tissue obtained from patients who underwent cystoscopy and/or transurethral resection of bladder tumor (TURBT) during 2008-2012. Blocks with >70% cancerous tissue were selected after histological examination of slides and categorized into non-muscle invasive low grade and muscle invasive high grade urothelial bladder cancer. In addition, 10 blocks of benign bladder mucosa with no evidence of malignancy were obtained from patients who underwent cystoscopy and bladder biopsy during 2007-2012 for different urinary complaints; these served as control (Figure 4). Approximately 5 ml of venous blood sample was also collected from 10 age matched healthy individuals and 16 patients with invasive high grade urothelial bladder cancer who underwent cystoscopy and/or TURBT in 2012.

## DNA extraction from FFPE samples

DNA was extracted from formalin-fixed paraffin embedded (FFPE) blocks using QIAamp® DNA FFPE tissue kit (Qiagen). Briefly, around 5-7 sections of 5-10 μm thickness were cut from block with microtome (Leica Inc.) and collected in an eppendorf tube. Tissue sections were deparaffinized by adding 1 ml xylene to the sample, tube was vortexed vigorously and then centrifuged at 14,000 rpm for 2 minutes at room temperature. Supernatant was removed and 1 ml of ethanol was added (to remove residual xylene from the sample), mixed by vortexing and centrifuged at 14,000 rpm for 2 minutes at room temperature. Supernatant was removed and the pellet air dried at 37°C until residual ethanol had evaporated. Tissue samples were homogenized in the Buffer ATL (180 μl) provided in the kit. Homogenized tissue samples were subjected to digestion by adding 20 μl proteinase-K at a concentration of 10 mg/ml, mixed by vortexing and tube incubated at 56°C for 1 hour to ensure complete tissue lysis. To reverse the formaldehyde modification of nucleic acids, tubes were incubated at 90°C for 1 hour. DNA was placed in a spin column, washed *in situ*, and eluted in 50 μl TE buffer. This DNA was quantitated by Nanodrop ND-1000 Spectrophotometer (Thermo Scientific). About 1-2 μg of DNA with A260:A280 > 1.8 was subjected to bisulfite modification using EpiTect® Bisulfite kit (Qiagen) following manufacturer’s protocol.

### DNA extraction from plasma

DNA was extracted from plasma samples using QIAamp® DNA Blood Mini kit (Qiagen) following manufacturer’s instructions. Briefly, 200 μl of plasma sample was added to a microcentrifuge tube containing 20 μl Proteinase K and was mixed thoroughly by pipetting. Lysis buffer AL (200 μl) was added to this mixture and incubated at 56°C for 10 minutes after thorough vortexing. The tubes were centrifuged briefly, and absolute ethanol (200 μl) was added to the sample, pulse vortexed for 15 seconds and centrifuged briefly. The entire mixture was applied to the QIAamp Mini spin column in a 2 ml collection tube (provided in the kit). The columns were centrifuged at 8000 rpm for 1 minute, flow through was discarded and columns were placed on to new collection tubes. Two washings were done with 500 μl buffer AW1 and AW2, filtrate was discarded and columns were placed on new collection tubes. QIAamp Mini spin columns were placed in a new 1.5 ml microcentrifuge tube, 200 μl buffer AE was added to the spin column and was incubated at room temperature (15-25°C) for 5 minutes, and then centrifuged at 8000 rpm for 1 minute to elute the DNA. The DNA samples were quantitated by Nanodrop ND-1000 Spectrophotometer (Thermo Scientific) and only samples with good concentration and nucleic acid/protein ratio > 1.8 were processed further to bisulfite conversion.

### Bisulfite conversion

Sodium bisulfite conversion of genomic DNA extracted from paraffin embedded blocks and plasma samples were carried out using EpiTect Bisulfite kit (Qiagen) as per manufacturer’s protocol. Bisulfite reactions were carried out in 200 μl PCR tubes on Mastercycler (Eppendorf) using the following conditions: 95°C for 5 min, 60°C for 25 min, 95°C for 5 min, 60°C for 85 min, 95°C for 5 min, and 60°C for 59 min.

Subsequently, purification of DNA was carried out by adding 310 μl of freshly prepared Buffer BL containing 10 μg/ml of carrier RNA and vortexing. Then 250 μl of ethanol was added and vortexed for 15 seconds. The entire mixture was transferred to EpiTect spin column and centrifuged at 14,000 rpm for 1 minute. Then 500 μl of Buffer BD (desulfonation buffer) was added and column was incubated for 15 min at room temperature. After washing by 500 μl of Buffer BW, DNA was eluted by addition of 20 μl of buffer EB and centrifuged at 14,000 rpm for 1 minute. Bisulfite converted DNA was stored at -20°C until use.

### Methylation-specific PCR

Promoter methylation analysis at *RASSF1A*, *APC*, *MGMT*, *CDKN2A* and *CDKN2B* was carried out with bisulfite converted DNA on Chromo-4 cycler (Bio-Rad) with Maxima SYBR Green Master mix (Fermentas) as the intercalating dye. Primer sequences are given in Additional file 1: Table S1. The following thermal cycling conditions were used: 95°C for 10 min, followed by 44 cycles of 95°C for 15 sec, and 60°C for 1 min. Melting curve was obtained from 65°C to 90°C.

Approximately 10 ng of methylated and bisulfite converted human DNA (Epitect® Control DNA, Qiagen) was used as control in which, complete in vitro methylation of the control DNA was achieved using *Sss*1 methylase and Bisulfite conversion of control DNA was achieved using the EpiTect Bisulfite kit. The housekeeping gene β-actin was used as the normalization control.

All samples were run in duplicate. Data was generated on Opticon Monitor 3 software (Bio-Rad). The software GENEX 5 was used to analyze the results. Samples with Ct value <35 were considered as positive.

### Normalized index of methylation

The Ct value of each gene was related to its copy number and Normalized index of methylation (NIM) was calculated as the ratio of copy number of gene of interest in the sample to that of control and both were normalized to the copy number of housekeeping gene i.e.;

Where, (Gene _Sample_) is the number of fully methylated copies of the gene of interest in a given sample, (Gene _Control_) is the number of fully methylated copies of the gene of interest in the *SssI* universally methylated control DNA, (*Actin*_Sample_) is the number of *Actin* copies in a given sample, and (*Actin*_Control_) is the number of *Actin* copies in the *SssI* universally methylated DNA.

### RNA extraction from FFPE samples

RNA was extracted from paraffin embedded blocks using RNeasy® FFPE kit (Qiagen) according to manufacturer’s protocol. Briefly 5-10 sections of 5 μm thickness were cut from the paraffin embedded blocks with microtome (Leica Inc.). After paraffin removal with xylene, samples were processed under denaturing conditions with proteinase K and incubated at 80°C for 15 minutes to reverse formalin cross-links. Genomic DNA was removed by passing the lysate through gDNA elimination spin column provided in the kit. Sample was placed in a spin column, washed *in situ*, and eluted in 30 μl RNase free water. Approximately 1 μg of RNA with A_260_/A_280_ ~ 2 was subjected to reverse transcription using Quantiscript reverse transcription kit (Qiagen) following manufacturer’s protocol. RNA expression of *RASSF1A*, *APC* and *MGMT* was determined by real time-PCR (Bio-Rad) using SYBR green using specific primers. Primer sequences are given in Additional file 2: Table S2.

### Statistical analysis

Statistical analysis was carried out using SPSS (version 19). The statistical tests used to evaluate the difference in the promoter methylation of candidate genes among normal bladder mucosa, non-invasive and invasive urothelial bladder cancer were Kruskal-Wallis and Mann–Whitney-U. Difference in the RNA expression between muscle invasive (high grade) and non muscle invasive (low grade) urothelial bladder cancer was evaluated using Wilcoxon signed rank test. Paired *T*-test was used to evaluate if there is any difference in the findings of promoter methylation status using paraffin embedded blocks versus blood samples. All p-values <0.01 were considered statistically significant. The association between the promoter methylation status and patients’ survival (Overall and recurrence free) was studied applying Kaplan-Meier survival plots.

## Electronic supplementary material

Additional file 1: Table S1: Primer sequences for the tumor suppressor genes included in the study (Xu et al. 2004). (DOC 29 KB)

Additional file 2: Table S2: Primer Sequences for RASSF1A, APC and MGMT real time RT-PCR analysis. (DOC 28 KB)

## Abbreviations

APC: Adenomatous polyposis coli
CDH1: Cadherin1
DAPK: Death associated protein kinase
FFPE: Formalin fixed paraffin embedded
FHIT: Fragile histidine triad
MGMT: O-6-Methylguanine-DNA methyltransferase
PTCH: Protein patched homolog 1
PTEN: Phosphatase and tensin homologue deleted on chromosome 10
RASSF1A: Ras- associated domain family 1A
RB1: Retinoblastoma 1
TSC1: Tuberous sclerosis 1
TURBT: Transurethral resection of bladder tumor.
