# Supplementary material for: Promoter hypermethylation of tumor suppressor genes correlates with tumor grade and invasiveness in patients with urothelial bladder cancer
Source: Springerplus. 2014 Apr 5;3:178. doi: 10.1186/2193-1801-3-178 (PMC4000596; doi:10.1186/2193-1801-3-178)
Supplement: Supplementary file 1 — Additional file 1: Table S1: Primer sequences for the tumor suppressor genes included in the study (Xu et al. 2004). (DOC 29 KB) [file 40064_2013_904_MOESM1_ESM.doc]

**Table S1- Primer sequences for the tumor suppressor genes included in the study (Xu et al. 2004).**

| **Gene** | **Methylated Primer Sequences** | **Size (bp)** |
| --- | --- | --- |
| *APC* | Fwd. 5’ TATTGCGGAGTGCGGGTC 3’  Rev. 5’TCGACGAACTCCCGACGA 3’ | 97 |
| *CDKN2A* | Fwd. 5’ TTATTAGAGGGTGGGGCGGGATCGC 3’  Rev. 5’ ACCCCGAACCGCGACCGTAA 3’ | 149 |
| *CDKN2B* | Fwd. 5’ GCGTTCGTATTTTGCGGTT 3’  Rev. 5’ CGTACAATAACCGAACGACCGA 3’ | 147 |
| *MGMT* | Fwd. 5’ TTTCGACGTTCGTAGGTTTTCGC 3’  Rev. 3’ GCACTCTTCCGAAAACGAAACG 3’ | 186 |
| *RASSF1A* | Fwd. 5’ GTGTTAACGCGTTGCGTATC 3’  Rev. 5’AACCCCGCGAACTAAAAACGA 3’ | 94 |
