# Supplementary material for: Promoter hypermethylation of tumor suppressor genes correlates with tumor grade and invasiveness in patients with urothelial bladder cancer
Source: Springerplus. 2014 Apr 5;3:178. doi: 10.1186/2193-1801-3-178 (PMC4000596; doi:10.1186/2193-1801-3-178)
Supplement: Supplementary file 2 — Additional file 2: Table S2: Primer Sequences for RASSF1A, APC and MGMT real time RT-PCR analysis. (DOC 28 KB) [file 40064_2013_904_MOESM2_ESM.doc]

**Table S2-Primer Sequences for *RASSF1A*, *APC* and *MGMT* real time RT-PCR analysis**

| **Gene** | **Forward Primer (5’->3’)** | **Reverse Primer (5’->3’)** |
| --- | --- | --- |
| **RASSF1A** | CGCGTCGTGCGCAAAGGCC | GGGTGGCTTCTTGCTGGAGGG |
| **APC** | GAGACAGAATGGAGGTGCTGC | GTAAGATGATTGGAATTATCTTCT |
| **MGMT** | GTGATTTCTTACCAGCAATTAGCA | CTGCTGCAGACCACTCTGTG |
